# The Impact of HIV and SSRI Treatment on Thrombocytopenia Risk

**DOI:** 10.1192/j.eurpsy.2025.1256

**Published:** 2025-08-26

**Authors:** A. Stewart, B. Carr

**Affiliations:** 1 College of Allopathic Medicine, Nova Southeastern Univeristy, Ft. Lauderdale; 2 Psychiatry, University of Florida, Gainesville, United States

## Abstract

**Introduction:**

Cytopenia is a common hematological issue in HIV/AIDS patients. Despite early disease identification and less toxic HAART reducing its prevalence, persistent thrombocytopenia remains. SSRIs, prescribed for depression in this population, can also cause thrombocytopenia. This review explores the potential combined impact of HIV and SSRI treatment on thrombocytopenia risk.

**Objectives:**

The objective of this study is to evaluate thrombocytopenia incidence in HIV/AIDS patients and those receiving SSRIs, and to provide clinical recommendations for monitoring in individuals affected by both conditions.

**Methods:**

A comprehensive literature review was conducted using PubMed, MEDLINE, and the Cochrane Library. Studies on thrombocytopenia in HIV/AIDS patients or discussing SSRI-induced thrombocytopenia were included. Data on incidence and clinical management were extracted and synthesized to form a cohesive understanding of risks and recommended practices.

**Results:**

Thrombocytopenia (<150,000 platelets/μl) is a sentinel event frequently seen in HIV/AIDS patients, often prompting further evaluation. A 1982 study reported thrombocytopenia in up to 40% of AIDS patients pre-HAART. Recent data from the CHORUS cohort (1997-2006) showed a decreased prevalence of thrombocytopenia to 14%. However, 23% of those with severe thrombocytopenia (<30,000 platelets/μl) remained symptomatic despite HAART. A BC-CfE study found a 0.6% prevalence of symptomatic thrombocytopenia among HAART-treated patients (1996-2012), highlighting non-HIV causes. SSRIs can cause thrombocytopenia by interfering with platelet serotonin uptake, reducing platelet function and lifespan. Several case series reported isolated thrombocytopenia following SSRI introduction. While the concurrent use of SSRIs and HAART’s effect on thrombocytopenia risk is unclear, careful consideration and monitoring are necessary.

**Conclusions:**

The relative risk of thrombocytopenia from the combined effect of HIV/AIDS and SSRI treatment has not been definitively established. HIV/AIDS patients on SSRIs should be carefully monitored due to the known hematological impacts of both the disease and medication. Regular monitoring of platelet counts is crucial, especially in those with advanced or poorly controlled HIV. Consider alternative psychiatric treatments with lower hematological risks where possible. Effective management requires interdisciplinary collaboration to address these patients’ complexities. This review underscores the importance of vigilant monitoring and individualized treatment strategies for HIV/AIDS patients on SSRIs to manage and mitigate the potential risk of thrombocytopenia.

**Disclosure of Interest:**

None Declared

